# Enhanced passive screening and diagnosis for *gambiense* human African trypanosomiasis in north-western Uganda – Moving towards elimination

**DOI:** 10.1371/journal.pone.0186429

**Published:** 2017-10-12

**Authors:** Charles Wamboga, Enock Matovu, Paul Richard Bessell, Albert Picado, Sylvain Biéler, Joseph Mathu Ndung’u

**Affiliations:** 1 Ministry of Health, Kampala, Uganda; 2 College of Veterinary Medicine, Animal Resources and Biosecurity (COVAB), Makerere University, Kampala, Uganda; 3 Epi Interventions Ltd., Edinburgh, United Kingdom; 4 Foundation for Innovative New Diagnostics (FIND), Geneva, Switzerland; Universidade Nova de Lisboa Instituto de Higiene e Medicina Tropical, PORTUGAL

## Abstract

**Introduction:**

The incidence of *gambiense* human African trypanosomiasis (gHAT) in Uganda has been declining, from 198 cases in 2008, to only 20 in 2012. Interruption of transmission of the disease by early diagnosis and treatment is core to the control and eventual elimination of gHAT. Until recently, the format of available screening tests had restricted screening and diagnosis to central health facilities (passive screening). We describe a novel strategy that is contributing to elimination of gHAT in Uganda through expansion of passive screening to the entire population at risk.

**Methodology / Principal findings:**

In this strategy, patients who are clinically suspected of having gHAT at primary health facilities are screened using a rapid diagnostic test (RDT), followed by parasitological confirmation at strategically located microscopy centres. For patients who are positive with the RDT and negative by microscopy, blood samples undergo further testing using loop-mediated isothermal amplification (LAMP), a molecular test that detects parasite DNA. LAMP positive patients are considered strong suspects, and are re-evaluated by microscopy. Location and upgrading of facilities to perform microscopy and LAMP was informed by results of georeferencing and characterization of all public healthcare facilities in the 7 gHAT endemic districts in Uganda. Three facilities were upgraded to perform RDTs, microscopy and LAMP, 9 to perform RDTs and microscopy, and 200 to screen patients with RDTs. This reduced the distance that a sick person must travel to be screened for gHAT to a median distance of 2.5km compared to 23km previously. In this strategy, 9 gHAT cases were diagnosed in 2014, and 4 in 2015.

**Conclusions:**

This enhanced passive screening strategy for gHAT has enabled full coverage of the population at risk, and is being replicated in other gHAT endemic countries. The improvement in case detection is making elimination of the disease in Uganda an imminent possibility.

## Introduction

Human African trypanosomiasis (HAT), also known as sleeping sickness, is a parasitic disease transmitted by the bite of an infected tsetse fly (*Glossina spp*). The disease is endemic in sub-Saharan Africa, within the limits of the geographic distribution of the tsetse fly. The disease is caused by protozoan parasites belonging to the species *Trypanosoma brucei*. Infection with *T*. *b*. g*ambiense* causes a chronic disease (*gambiense* HAT or gHAT) and accounts for more than 95% of HAT cases reported annually [1,2]. Infection with *T*. *b*. *rhodesiense* results in an acute form of HAT (*rhodesiense* HAT or rHAT). The two forms of disease are geographically separated, and Uganda is the only country that is endemic for both forms, albeit in geographically separate foci.

The number of HAT cases reported globally has been falling steadily, and consequently, gHAT has been included by the World Health Organization (WHO) in a roadmap for elimination as a public health problem by 2020 [3]. This WHO goal was endorsed by the London Declaration of 2012 [4]. However, to achieve elimination, novel tools and strategies are required, in order to ensure that the population at risk of infection is adequately covered by an intensive and sustainable surveillance system [5].

Control of gHAT primarily relies on identification and treatment of cases [6]. This is done through passive screening–where people present themselves to health facilities, and active screening–mass screening of communities that are at risk. In recent years, control of gHAT has been complemented with vector control [7]. Passive screening is hampered by lack of distinguishing clinical signs, and once they appear, they are similar to those of malaria, a disease that is endemic in all regions where HAT occurs. When the disease has advanced to a neurological form, the clinical signs are more distinct–characterised by sleep and behaviour disorders. Patients with such symptoms and are negative with a malaria test, or are unresponsive to malaria treatment, should be considered potential HAT cases and screened with a serological test for HAT. Until recently, the principal screening test for gHAT was the card agglutination test for trypanosomiasis (CATT), which requires cold storage and electricity, and is packed in multiple doses that are not optimal for screening individual patients [8]. This made it difficult to use the test in rural health facilities. Serological tests for gHAT do not have perfect specificity, and as treatment is associated with adverse events, a positive serological test must be confirmed by demonstration of parasites in body fluids (blood, lymph node aspirate or cerebrospinal fluid) by microscopy [9].

The potential for screening for gHAT in health facilities has been improved with the recent development of rapid diagnostic tests (RDTs) [10–13]. The tests are affordable and packaged for single use, do not require electricity, and can be stored at 40°C for up to two years [13], making them appropriate for use in any health facility in gHAT endemic regions.

Confirmatory diagnosis of HAT has been improved following development of the Primo Star iLED fluorescence microscope (FM) by FIND and Carl Zeiss Microimaging [14]. Unlike classical fluorescence microscopes, this microscope does not require a dark room, can be powered using small solar panels, and the light source lasts for more than 10,000 hours. A number of techniques for preparing and staining samples with acridine orange, then examining them using the LED FM have been developed [14,15]. This has been shown to improve sensitivity when used alongside existing techniques such as gland puncture (GP), and the microhaematocrit centrifugation technique (mHCT, CTC or Woo test), that are routinely used to confirm HAT [16]. The more sensitive mini-anion exchange centrifugation technique (mAECT) [17] had not been in routine use in Uganda prior to 2016. All these techniques require a small laboratory and staff that are trained in microscopy.

Diagnostic techniques for gHAT that are based on microscopy have imperfect sensitivity [15,18]. Detection of parasite DNA by a loop-mediated isothermal amplification (LAMP) technique has increased the prospects of reducing the proportion of missed cases by using molecular diagnostic tools [19–21]. However, the equipment used to perform the test requires an uninterrupted power supply, and is therefore only suited for larger and well equipped laboratories. However, LAMP can be performed on blood or buffy coat samples that are either fresh or after being dried and stored on filter papers [19].

**Fig 1:**
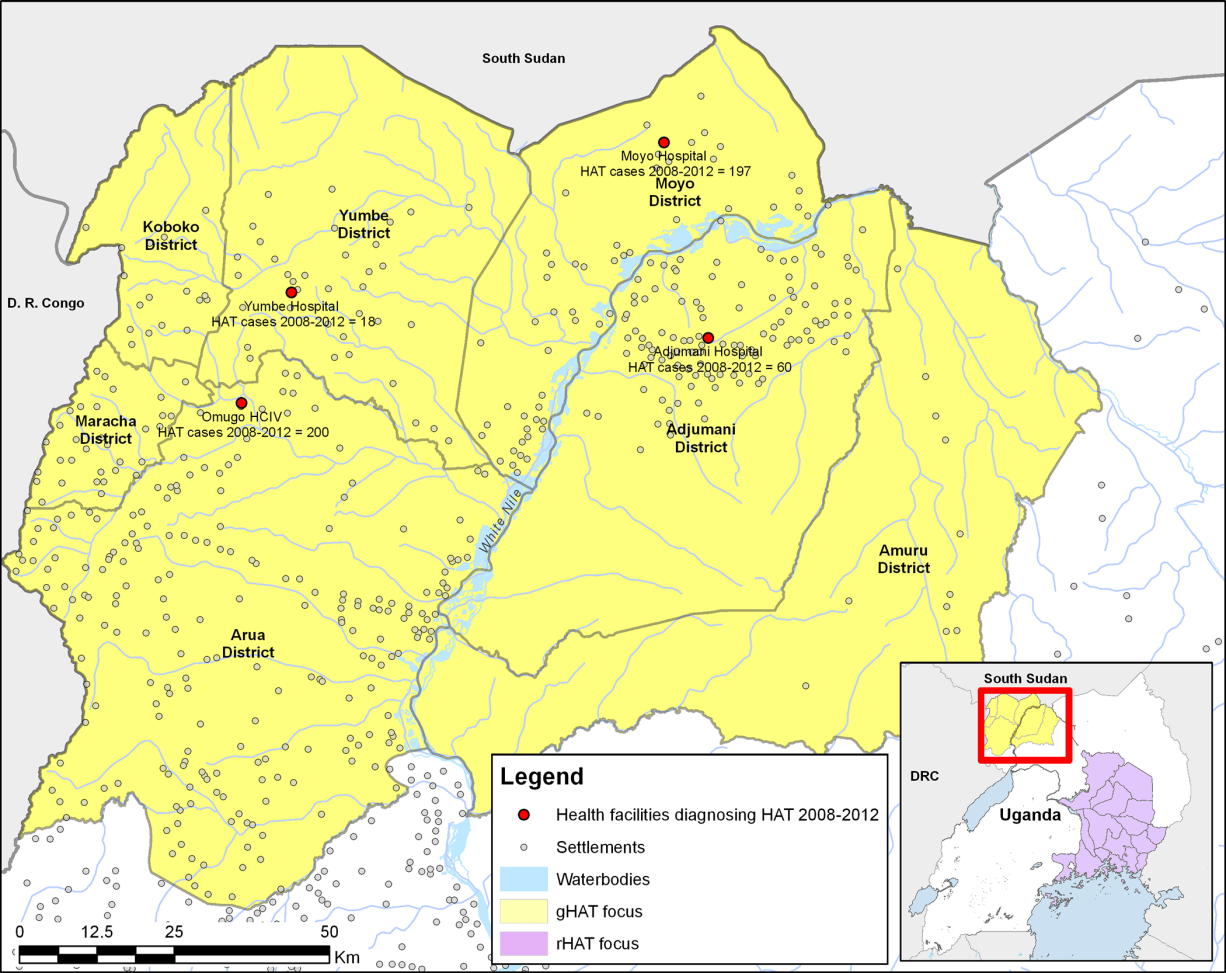
The gHAT endemic region in north-western Uganda, showing the 4 health facilities that performed confirmatory diagnosis of the disease prior to 2013, and the cases detected between 2008 and 2012. The gHAT region is geographically separated from the rHAT region. The geographic layers were obtained from CC-BY License compatible sources: GADM (http://www.gadm.org), RCMRD geoportal (http://geoportal.rcmrd.org/) and GeoNames database (http://www.geonames.org/).

**Fig 2:**
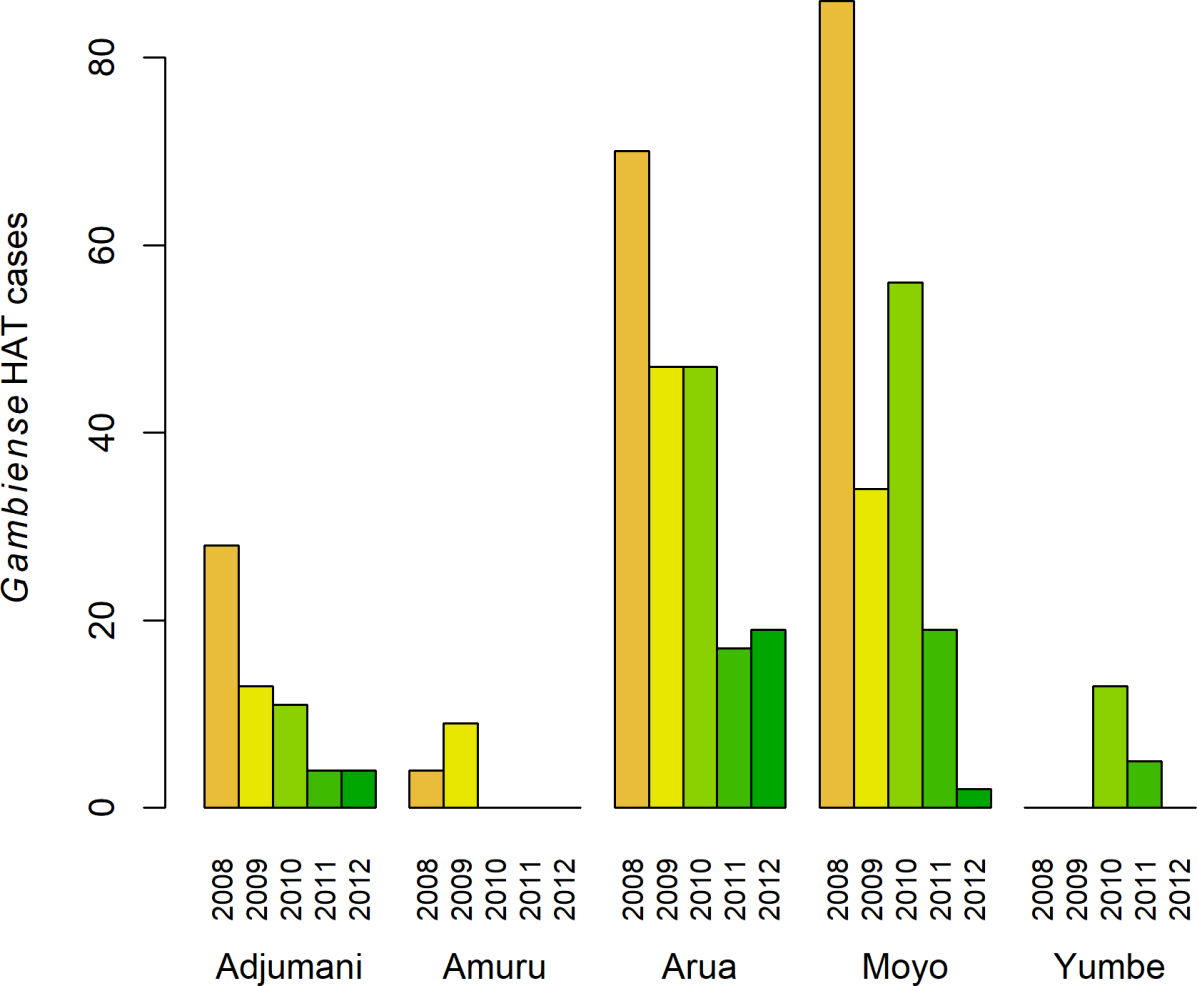
Barplot of gHAT cases reported in Uganda from 2008 to 2012, by district in which the case was diagnosed. Cases from the districts of Koboko and Maracha were referred to Arua for diagnosis and treatment.

In Uganda, the number of gHAT cases has been declining, with 198 reported in 2008 and only 20 cases in 2012 [22]. However, to ensure elimination of gHAT, all remaining cases must be swiftly identified and treated in order to remove their reservoir status. Increasing the number of facilities conducting passive screening for gHAT in the endemic region would ensure full coverage of the population at risk, making diagnostics readily accessible to any infected and sick patients. At the start of 2013, only four health facilities in the region could diagnose gHAT, and screening was performed using CATT. The 4 facilities, which are also the treatment centres, were serving a population of 2.22 million people [23] (Fig 1). In this region, 63% of the population at risk were living more than 1 hour away from a facility with gHAT diagnostic capacity, and although 56% of those at high and very high risk of gHAT were within 1 hour, 27% among this category were 3 or more hours from a facility ([24]—supplementary information). From 2008 to 2012, the largest number of cases were from Arua district (n = 200), and were confirmed at the Omugo Level 4 (HCIV) health centre (Fig 2). This was followed by Moyo district, where 197 cases were identified.

Here, we describe a programme that harnesses the recently developed technologies for screening and diagnosis of gHAT. This is deployed in the healthcare infrastructure in north-western Uganda to intensify control of the disease. We describe the approach used to identify and characterize health facilities in the region, selection of facilities and their upgrading, and how progress of the programme has been monitored.

## Methods

### Characterisation of health facilities

The first stage of the programme was to map the locations of all public and private not for profit (typically faith-based) healthcare facilities in the gHAT endemic region of Uganda, and to characterise them. The process, completed in 2012, involved compiling a list of all facilities in the districts, then visiting and recording their locations using a hand-held GPS. A questionnaire (supplementary information S1 Table) was completed with information on the health facility, including the population served by the facility, staff capacity and their levels of training, status of the laboratory, reagents, available materials, and the history of diagnosis of gHAT (if the facility had performed diagnosis of gHAT in previous years). The information generated was used to make a map showing the locations of the health facilities, relative to the road and river networks. In 2015, private for-profit clinics in the region and facilities that are well situated to serve refugees from South Sudan, were also characterized using the same methodology.

### Upgrading of health facilities and training

The map generated from the characterization data was used to identify strategically located health facilities that could be upgraded to offer confirmatory diagnosis of gHAT, with the aim of reducing the distance that a patient suspected of having gHAT would have to travel for confirmatory testing. Facilities that were well maintained, and had laboratory personnel, were upgraded and equipped to perform parasitological confirmation of gHAT using LED FM (the equipment is described in supplementary information S2 Table). Upgrading of facilities included installation of solar power in all those performing microscopy. Among these facilities, selected laboratories were also equipped to perform LAMP. Technicians in all the microscopy laboratories were trained to perform parasitological diagnosis of gHAT, and those in facilities that were not equipped with LAMP were also trained to dry blood samples onto filter papers, and store them before shipment to the LAMP facilities. Additionally, technicians in LAMP facilities were trained in LAMP, and supplied with a dedicated motorcycle, to be used in collecting samples from the microscopy laboratories, distribution of RDTs, and programme support in general. All health facilities in the entire project area were supplied with HAT RDTs, and health workers trained in their use. They were also trained in clinical diagnosis of gHAT, and on a new algorithm of passive screening and confirmatory diagnosis of the disease. Treatment of all gHAT patients continued at four facilities–Omugo HCIV, Yumbe Hospital, Moyo Hospital and Adjumani Hospital.

Alongside the upgrading of heath facilities, a community sensitisation campaign was carried out, including community barazas (meeting places), meetings with leaders at all levels, and radio broadcasts during the first year of the programme. Sensitisation focussed on informing the population of the symptoms of gHAT, availability of screening tests in local health facilities, and the process of diagnosis and referral.

### Diagnostic procedure

**Fig 3:**
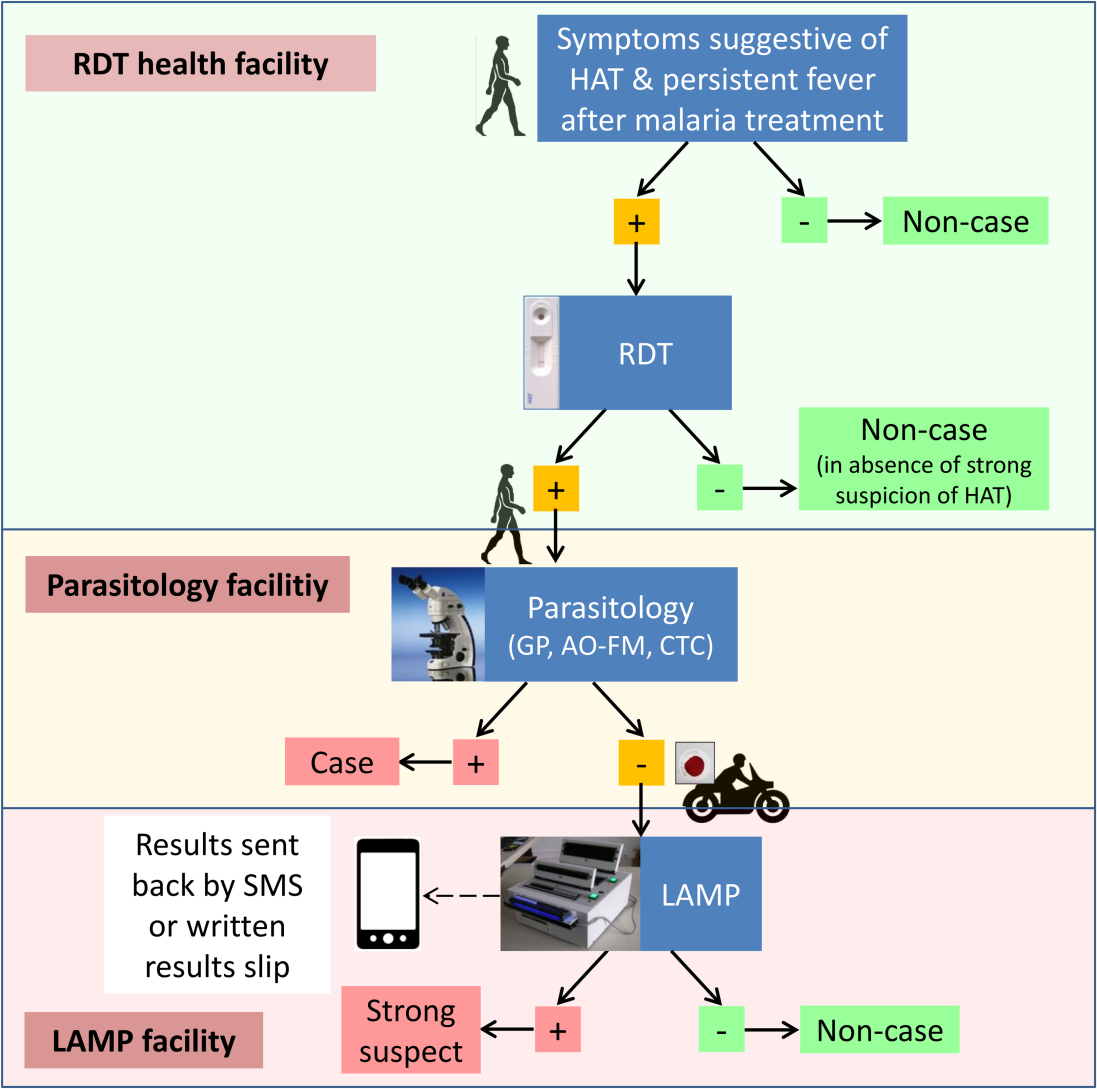
The *T*. *b*. g*ambiense* human African trypanosomiasis (gHAT) diagnostic workflow implemented in Uganda. Gland puncture (GP); Acridine Orange–Fluorescence Microscopy; capillary tube centrifugation (CTC or mHCT).

The diagnostic procedure for gHAT is initiated when a patient presenting at a health facility is found to have symptoms that are suggestive of gHAT (symptoms suggestive of gHAT include speech disorders, enlarged cervical glands, behavioural disorders, walking disorders, sleeping disorders, convulsions or epilepsy and fever). If symptoms that are also compatible with malaria are observed, then the patient is first tested with a malaria RDT, and if found to be either: negative for malaria, or positive for malaria but not responding to treatment after one week, then the patient is tested using the HAT RDT. If the symptoms are not consistent with malaria, the patient is tested with the HAT RDT. Patients found positive with the HAT RDT are considered as gHAT suspects, and are referred to the nearest facility equipped for parasitological confirmation, unless the HAT RDT is performed at such a facility, in which case no referral is necessary (Fig 3). Patients who are negative by the HAT RDT undergo investigations for other diseases that could present similar symptoms.

Parasitological diagnosis entails performing a number of tests in the following sequence, until one of them is positive:

- If the subject shows typical cervical adenomegaly, a GP is taken and examined for the presence of motile parasites by bright field microscopy.
- If no palpable lymph nodes are present, then 7ml of venous blood are collected and the following tests performed:
  ○ The mHCT test is performed using four capillary tubes [16].
  ○ A thick blood smear is made on a microscopy slide using 5μl of blood, stained with Acridine Orange (AO), and examined by LED FM as described elsewhere [14].
  ○ 3 ml of blood are used to perform the red blood cell (RBC) lysis and concentration method, followed by staining with AO, and examination by LED FM, as described elsewhere [14].

When a patient is negative by microscopy, a minimum of 4ml blood is centrifuged and the buffy coat separated. The buffy coat is lysed with sodium dodecyl sulfate (SDS) solution, and in facilities without the capacity to perform LAMP, 100μl is dried on a filter paper and kept in an air-tight plastic envelope with a desiccant [25]. Similarly, 100ul of whole blood is also dried on a second filter paper. The dried samples are transported by motorcycle to the nearest LAMP centre and tested within 14 days from the date of collection. At the LAMP centre, both samples are tested for presence of parasite DNA using LAMP according to the standard operating procedure [25].

If the patient is at a facility that has the capacity to perform LAMP, a minimum of 4ml of blood is used to perform LAMP on fresh whole blood, and on fresh buffy coat that has been extracted by centrifugation followed by treatment with SDS solution. If any of the two samples is positive by LAMP, then the overall result is interpreted as positive. The results are communicated by SMS to the facility that performed the RDT, and when positive, the patient is requested to come back and parasitological testing is repeated. For all confirmed gHAT cases, the stage of disease is determined by performing a lumbar puncture and examination of cerebrospinal fluid by microscopy, then they are treated at one of the treatment facilities, in accordance with the national guidelines.

Patients who are positive with the HAT RDT but negative by both microscopy and LAMP are followed up and tested every three months with the HAT RDTs until they become seronegative, or are confirmed as cases. If a patient is still positive by the HAT RDT during follow-up, then the procedures described above are repeated.

### Data collection and analysis

Data on RDT usage, stock levels, number of positive patients and numbers referred, are recorded for each facility, then collated at the district level and transmitted to the central level by mobile phone. These data are subsequently collated and are analysed to assess progress and identify potential problems or unusual patterns in RDT usage, RDT positivity or HAT cases. Data are analysed monthly, and any remedial action found to be necessary is taken immediately.

Access to screening facilities was analysed by calculating a raster surface of the Euclidean distance to all facilities. Adjusted estimates of the human population for 2015 from the WorldPop dataset [26] are overlain on the distances. Access to screening by the population was calculated by weighting the distances to facilities by the population and analysed using cumulative distribution plots using the ecdf() function in the R statistical environment [27]. Access to confirmation (diagnosis was calculated using the Euclidean distance between screening and the closest diagnostic centre and illustrated using density with the density() function in R [27] at the default settings. In these calculations we assume that people will not cross river Nile in seeking diagnosis, so it is the nearest diagnostic site on the same side of the river.

### Number of facilities conducting passive screening for gHAT

The programme is responsive to the changing epidemiology and diagnostic needs in the region. The number of people found positive with HAT RDTs and their location, as well as the origin of gHAT cases identified in the 12 previous months were used to re-assess the number of health facilities included in the programme. The number of health facilities using HAT RDTs were reviewed in July 2014 and September 2015, in response to the evolving epidemiological situation of gHAT in Uganda, particularly in relation to the large numbers of refugees arriving from gHAT endemic areas of South Sudan [28].

### Ethical approval

This project is carried out in conformity with the Helsinki Declaration. The project protocol was reviewed by Vector Control Division-Research Ethics Committee, Uganda Ministry of Health, and approved by the Uganda National Council for Science and Technology (registration number HS 1427). The project sites have the necessary facilities and trained staff to test patients and collect samples under GCLP conditions. Although the diagnostic methods in use (RDT, LED FM and LAMP) have been rigorously evaluated in multiple sites, patients are requested to give informed consent at the level of the microscopy or LAMP centre, as LAMP is still an investigational test.

## Results

### Programme initiation

**Fig 4:**
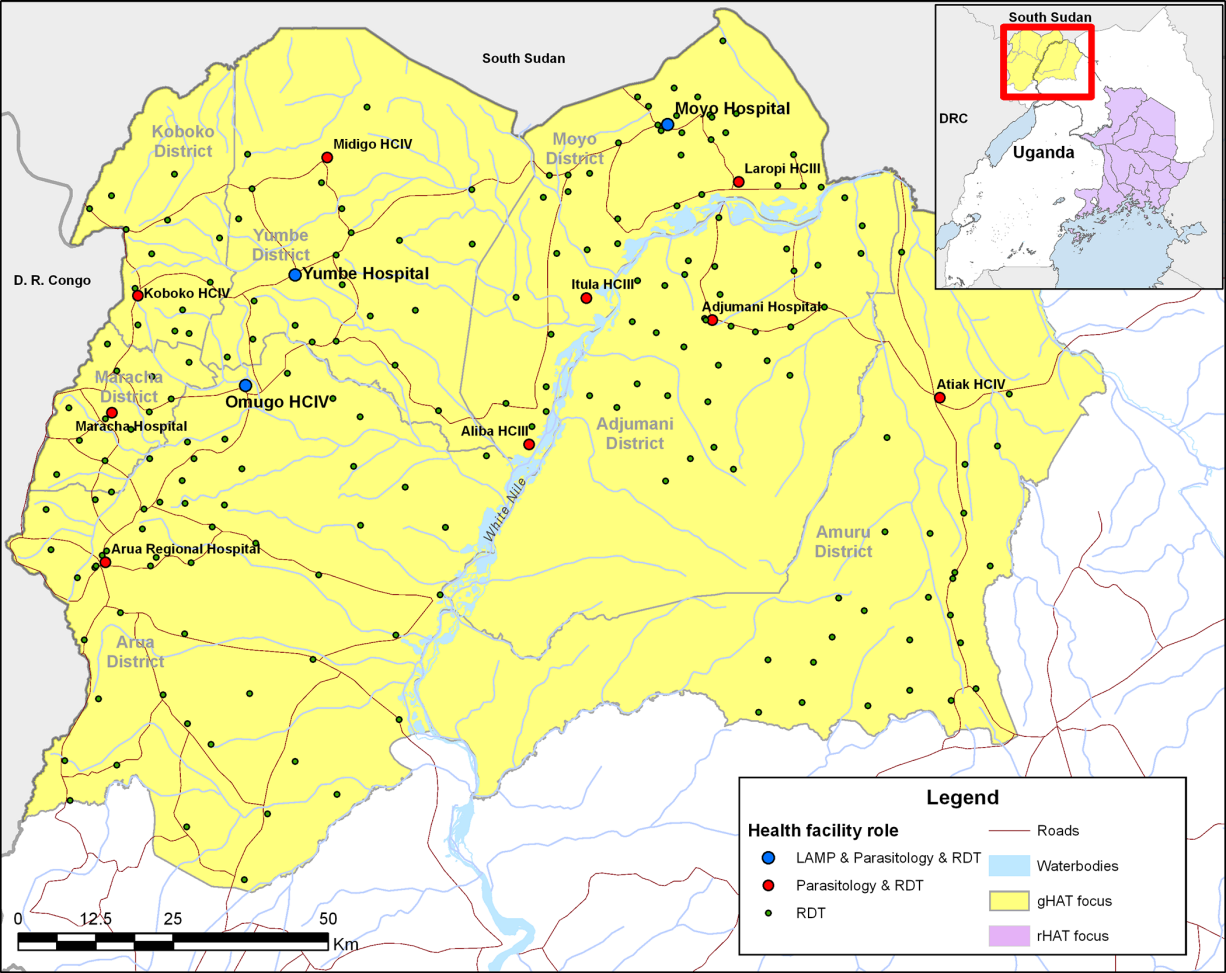
Map showing health facilities in the gHAT focus of north-western Uganda that were characterised and equipped to perform RDTs, microscopy and LAMP in the initial phase between May 2013 and February 2014. HCIV = level four health centre; HCIII = level 3 health centre. See details on the sources of the geographic data in Fig 1.

Two hundred and ten facilities were characterised, comprising 116 level II health centres (the lowest level of government health facilities), 78 level III health centres, 9 level IV health centres, 6 district hospitals and 1 regional referral hospital. Characterisation of facilities commenced in July 2011, and was completed in December 2011. These 210 and two others that were not characterised were equipped with HAT RDTs, with appropriate training in use of the RDTs and the diagnostic algorithm. Among these 212 facilities, 9 were also equipped to perform microscopy, and a further 3 to also perform microscopy and LAMP (Fig 4). Technicians in all the microscopy and LAMP facilities received appropriate training. These activities were carried out between May 2013 and January 2014.

**Fig 5:**
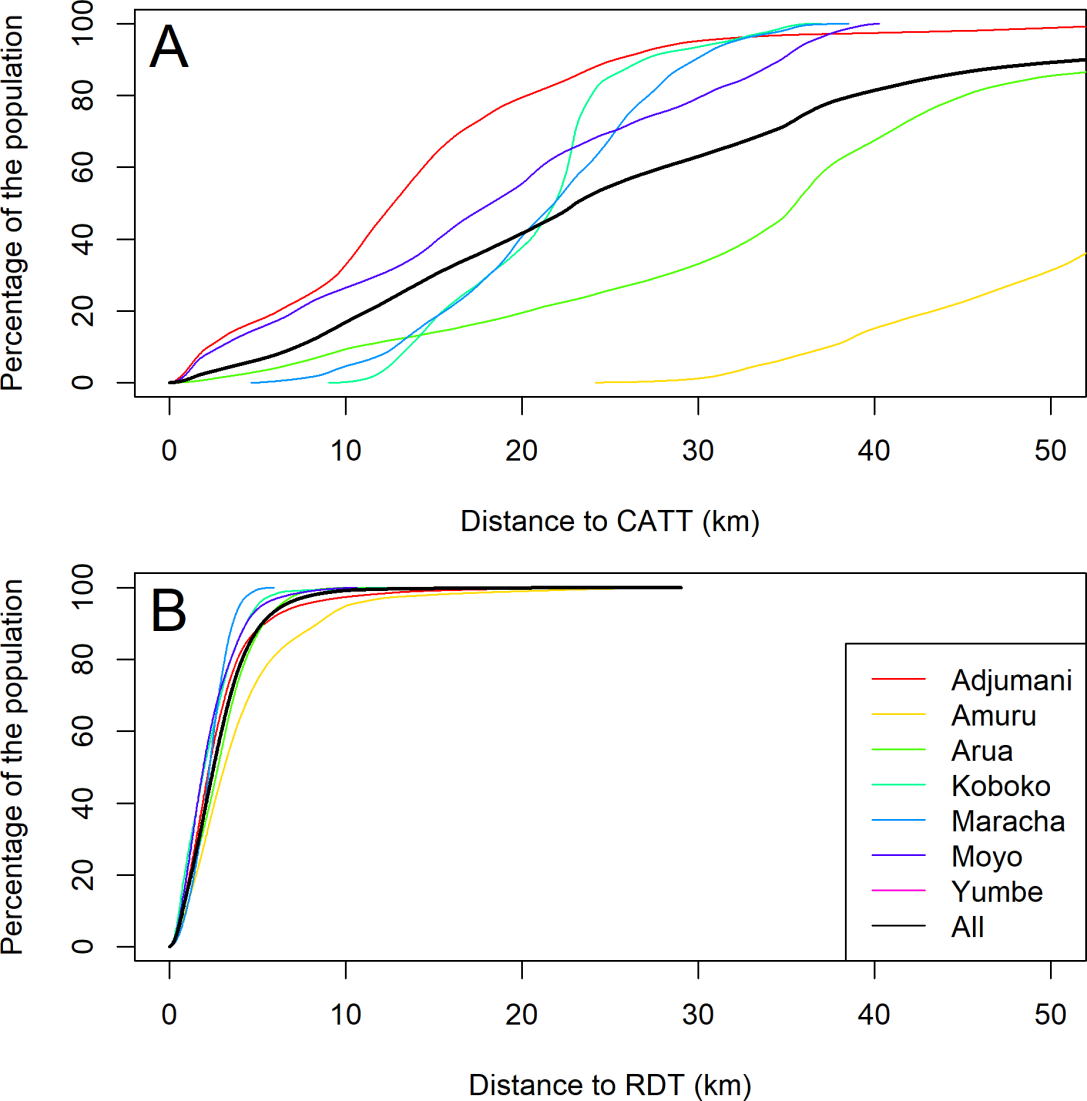
Cumulative distribution plots (as lines) of the distance of the population to a screening test. The calculation of cumulative distribution is such that for each distance location on the x-axis, the corresponding percentage on the y-axis are within that distance. This is illustrated by district (A) prior to expansion of screening and (B) after expansion of screening. Accordingly, considering all districts, 50% of the population were within 23km of a screening facility prior to this programme, compared to 2.5km following the programme.

The increase in number of screening facilities reduced the median straight line distance that the population at risk must travel for screening, from 23km to 2.5km, with 99% of the population within 9.6km of a facility that is equipped with RDTs (Fig 5). This remains the case when we consider Adjumani—the district that was best served by CATT from Adjumani Hospital, where the median distance was reduced from 12.7km to 2.2km (Fig 5).

**Fig 6:**
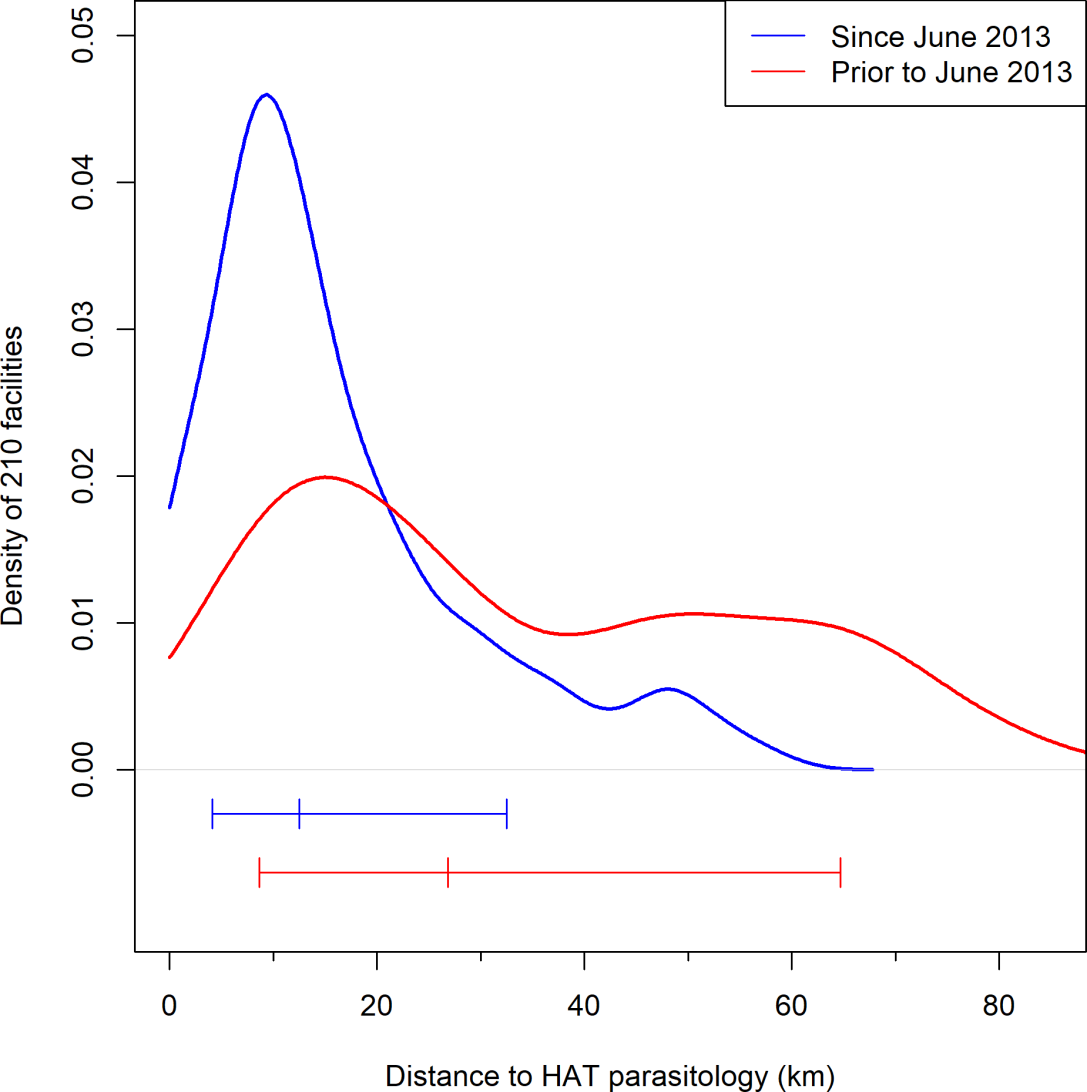
Density plot of the Euclidean distance to gHAT diagnostics (microscopy) for the diagnostic coverage prior to this programme and under this programme. The top lines are density plots and the lines below represent the median and central 75% of the data. Density plots were calculated using a Gaussian kernel with a bandwidth of 3.17km. Note that this was calculated separately for the facilities on the west and the east of River Nile, assuming the Nile to be a barrier that would not be crossed.

The higher density of microscopy facilities that were established along the borders with the Democratic Republic of the Congo (DRC) and South Sudan reflects the historically larger number of cases in this area compared to the southern areas of Arua district, as well as in Adjumani and Amuru districts to the east of River Nile (Fig 5). The median straight line distance (allowing for River Nile as a barrier) from an RDT facility to a facility performing microscopy is 12.49km, compared to 26.8km from a facility performing HAT screening to a facility performing microscopy prior to the study (Fig 6). The median distance from a microscopy facility to a LAMP facility is 22.23km.

### Progress of project to December 2015

**Fig 7:**
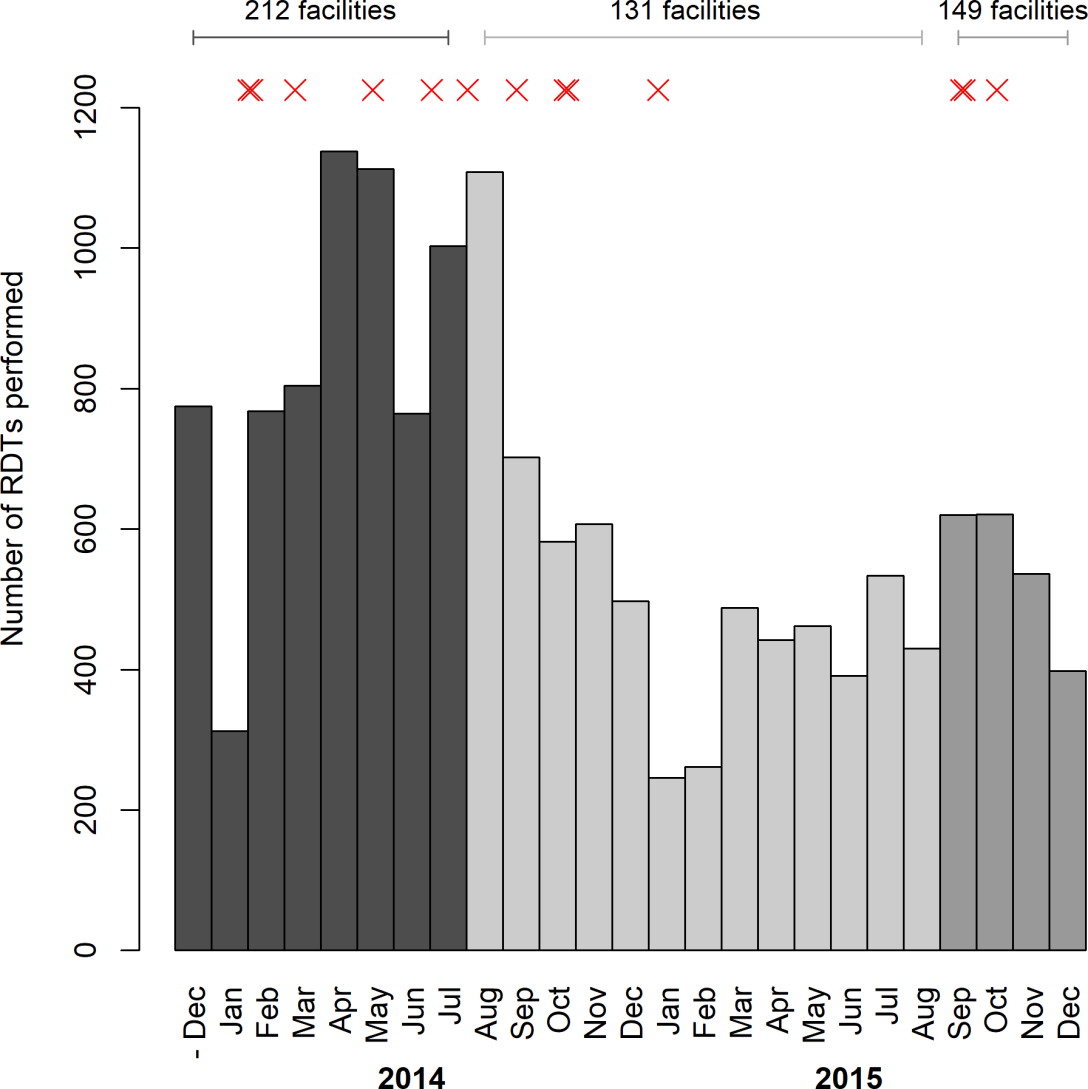
Usage of HAT RDTs by month of the programme. A red ‘X’ represents a gHAT case that was confirmed. The decline in numbers after August 2014 corresponds with the reduction in the number of health facilities using RDTs in Amuru and Adjumani districts, and the increase in September 2015 corresponds to the expansion to private clinics and re-engagement of refugee facilities.

By 31^st^ July 2014, 6,677 RDTs had been performed (mean of 0.175 RDT per facility per screening day; range 0.007–0.73) (Fig 7). During this time, 200 patients were positive with RDTs (3.0% RDTs positive), and 79.9% of positive RDTs from RDT facilities were tested by microscopy. 9,790 RDTs were performed from August 2014 to December 2015. Thirteen gHAT cases were identified between programme initiation and December 2015 (9 in 2014 and 4 in 2015). All cases were in late stage disease. Eight (61%) were confirmed by microscopy (2 by GP; 1 by GP and LED FM; 5 by mHCT) at first presentation. Four of the cases (31%) were identified in the second parasitological test (all by mHCT) following a positive LAMP result. Finally, one case (8%), initially RDT negative, was positive by LAMP and confirmed by lumbar puncture as the patient presented symptoms highly suggestive of gHAT. Of those that were RDT positive but were not confirmed by microscopy, 61 had been followed-up at the time of writing, but none had resulted in a diagnosis of gHAT.

**Fig 8:**
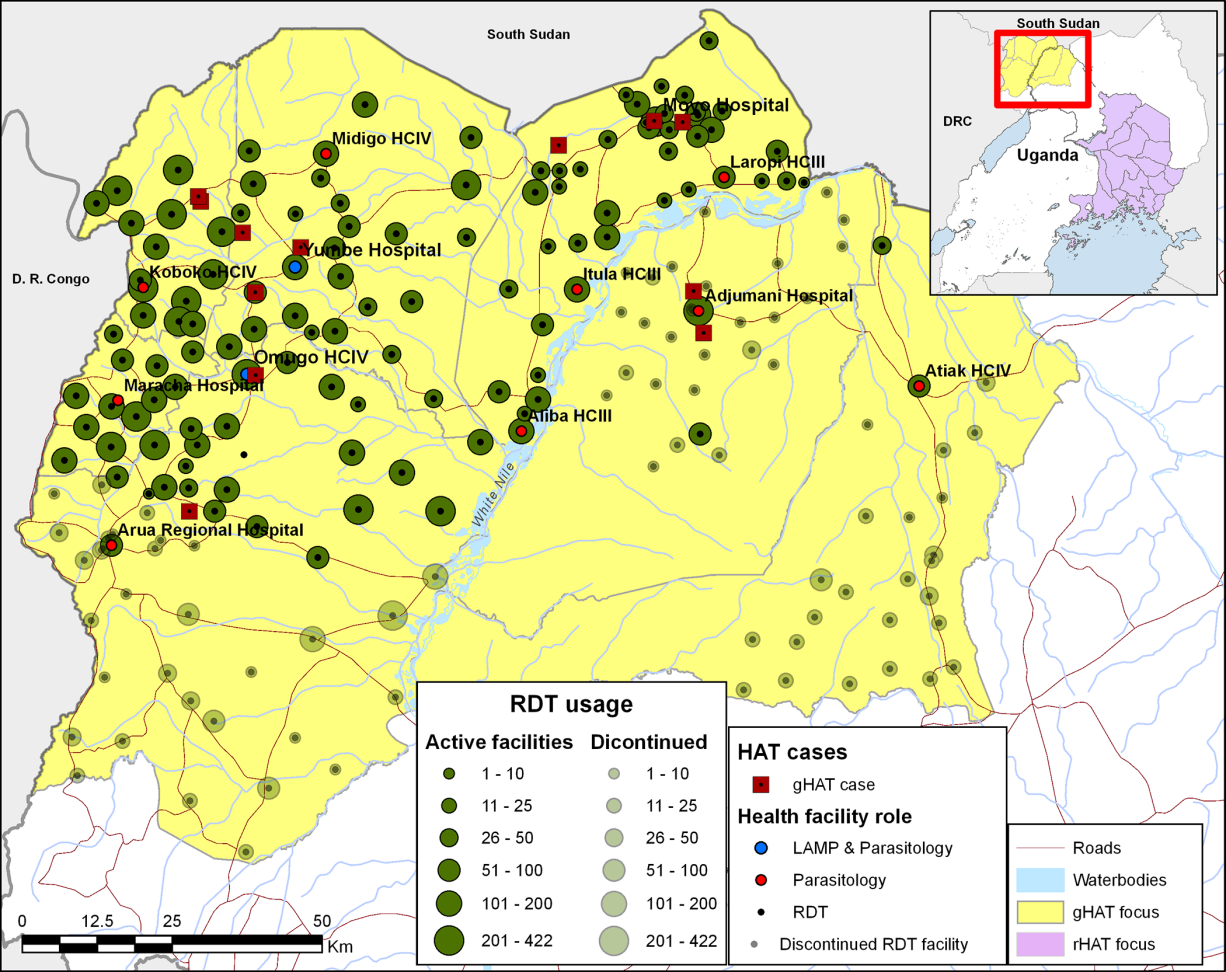
Usage of HAT RDTs by facility between August 2013 and December 2015. Note that one gHAT case was from South Sudan and is outside the map extent. The case locations correspond to the home village of the cases. See details on the sources of the geographical data in Fig 1.

The 13 HAT cases were spread across the project area, with one case originating from South Sudan (Fig 7). Of these cases, 3 were identified at health facilities that were not previously screening for gHAT, including one private clinic that was enrolled in September 2015. Relatively few RDTs were used in the districts of Adjumani, Amuru and southern areas of Arua district (20.9% of the total RDTs were performed in 88 facilities in these areas) and, as a consequence, activities were scaled back in these areas–leaving just four sentinel facilities. At the same time, 3 additional public facilities in Yumbe district were enrolled. As a consequence of the drop in numbers of participating facilities, there was a drop in the monthly rate of usage in RDTs in the period from July 2014 to December 2015, with 9,790 RDTs performed in the period from August 2014 to December 2015 (Fig 7). The majority of the RDTs were performed in the north around Moyo, and in the west in Maracha, Koboko and Yumbe districts (Fig 8). Case identification continued, with 5 cases identified prior to scale-back and 8 following scale-back (Fig 7). In September 2015, 16 private clinics in Koboko and Yumbe towns were included in the project, and in Adjumani district, 3 facilities serving refugees that had been dropped were re-engaged along with two other facilities.

**Fig 9:**
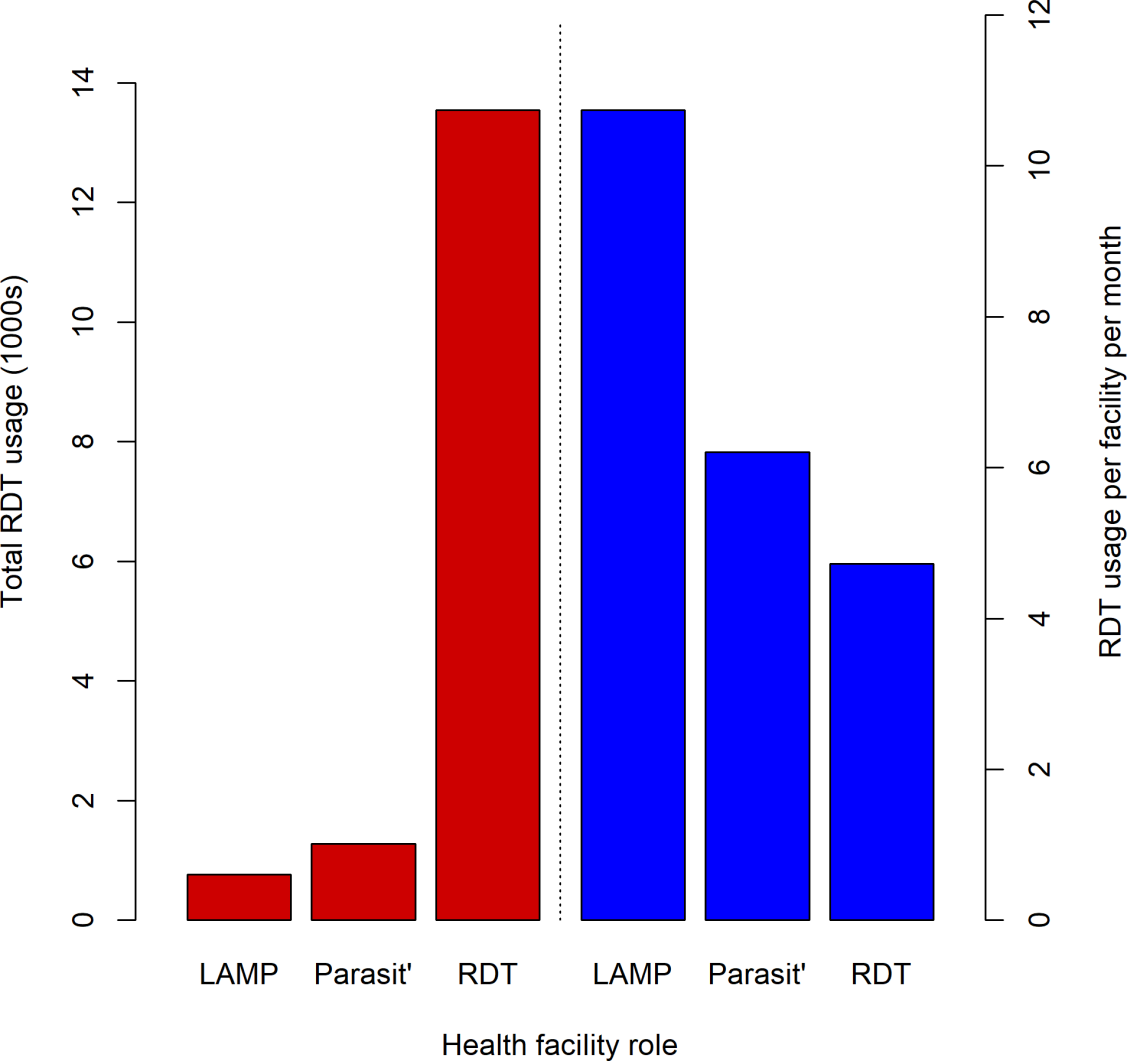
Barplot of total HAT RDT usage to December 2015 (A) and monthly rate of RDT usage (B), by type of facility. Parasit’ = parasitology.

The majority of the HAT RDTs were used in RDT facilities owing to their larger number. However, RDTs were used at a higher rate at facilities with LAMP and microscopy than by facilities with only RDTs, where they were used at a mean rate of 4.72 RDTs per month, compared to 10.7 and 6.2 RDTs per month in facilities with LAMP & microscopy and microscopy respectively (Fig 9).

## Discussion

We have described the development and implementation of a new strategy for enhanced surveillance and diagnosis of gHAT. As elimination of gHAT as a public health problem in Uganda and elsewhere draws nearer, novel strategies are required to guarantee that the goal of elimination is achieved and sustained in an arena with limited financial resources. This has been made possible with the development of new diagnostic and screening technologies that, with modest capital investment, have opened up the potential of integrating gHAT surveillance and control into the state primary healthcare system.

Although this strategy has resulted in an increased number of gHAT suspects identified by passive screening, the number of gHAT cases in Uganda has continued to decline, with just 9 cases in 2014 and 4 cases in 2015, a decrease from 20 that were diagnosed during 2013. In 2016, the number of gHAT cases in Uganda remained at four. This decline could be attributed to multiple factors, including change in land use or the large scale vector control activities in areas to the west of River Nile, which resulted in a drop in tsetse fly densities of 90% [7,29]. By increasing coverage of screening for gHAT, it is likely that fewer cases go undetected and untreated. Since these untreated cases contribute to the reservoir of gHAT, identifying and treating a greater proportion of cases decreases the overall disease transmission [30–32]. The combination of enhanced passive screening and vector control could be further complemented by other control strategies, such as reactive active screening, in which people in villages are screened by small mobile teams in response to identification of one or more cases. However, at the core of this is a strategy that ensures that clinical suspects can be screened closer to their homes, and that access to confirmatory diagnosis is improved.

By upgrading 12 facilities to perform confirmatory testing for gHAT, there has been a substantial reduction in the distance that is necessary to travel to get a diagnosis for gHAT. Previous studies of rHAT in Uganda have demonstrated that difficulties in accessing appropriate diagnostics can be a barrier to diagnosis [33–35] and this is known to be a barrier throughout HAT endemic regions [24,36]. Further delays can be introduced by patients requiring a median of four visits to heath facilities and a seven months’ delay before being screened for gHAT [37]. Sensitisation of the public and health personnel was a core component of this new strategy. As case numbers decline, and community awareness of HAT wanes, the importance of vigilance among local healthcare providers increases, if the remaining cases are to be identified. By improving access to diagnostics to include all health facilities, and increasing knowledge and awareness of gHAT, the strategy described here minimises delays to diagnosis and treatment [38].

Confirmation of gHAT cases remains complex, and one of the challenges of this novel approach is ensuring that patients that are positive by an RDT at a facility without capacity for microscopy present themselves for confirmatory testing. In the new strategy, the patient is referred to another facility where, owing to the low prevalence and the resulting low positive predictive value of screening tests, the final outcome is often negative. To mitigate this, sensitisation was conducted at all levels, to change expectations among patients and health personnel, and to implement the new strategy to ensure that patients do complete their referral. Consequently, the proportion of RDT positive individuals tested by microscopy in our project was high (79.9%). During the course of the programme this has been complemented by the active follow-up of serological suspects at three month intervals until they become negative, and by active follow-up of those that did not present for parasitological confirmation by motorcycle teams.

The diagnostic algorithm described here required training of staff at health facilities, many of whom were not familiar with HAT as a differential diagnosis. However, as the project area (like all HAT foci) is also endemic for malaria, staff at all facilities were already familiar with performing malaria RDTs, which are done in the same way as gHAT RDTs. In spite of familiarity with the RDT format, it was necessary to keep health staff sensitised to consider gHAT in their differential diagnosis [39], which is monitored through continuous tracking of data on usage of HAT RDTs in the health facilities. It was also critical to train health personnel on how to interpret RDT results and communicate them to patients in order to achieve satisfactory referral. It is interesting to note that following expansion of the screening coverage, the drop in the numbers screened at the four facilities that provided screening prior to the expansion was small, and was not commensurate with the 53-fold increase in coverage (S1 Fig). This indicates that there has been a change in the awareness of clinicians around the disease, or willingness to screen people with the single format test.

The new diagnostic algorithm described here is integrated in the primary health system in rural Uganda. Thus, HAT screening has become part of the routine activities in health centres in endemic areas. Implementation of the algorithm is also highly adaptable. It is used in private clinics that can refer RDT positive individuals to public facilities for confirmation. It can also be deployed in less or more facilities depending on the epidemiological circumstances. For example, the number of centres using HAT RDTs was increased in 2016 to respond the new challenge of refugees fleeing the armed conflict in South Sudan and seeking refuge in HAT endemic areas in northern Uganda [28]. HAT RDTs are now used in health facilities serving the refugee camps in Adjumani and Yumbe. The algorithm can also be modified to include new tests if required, such as a more sensitive confirmatory test: mAECT [17]. *T*.*b*. *rhodesiense* HAT is endemic in another region of Uganda, and there has not been any report of this form of the disease in the region described here. In the unlikely event that a *T*.*b*. *rhodesiense* HAT patient presented at any of the health facilities in the project area, he/she would likely be missed by the diagnostic work-flow described here, unless they presented with clinical signs that are strongly suggestive of HAT. In such situations they would undergo testing by microscopy, regardless of the result of the screening test.

In this project, LAMP is used as a ‘back-stop’ to the imperfect sensitivity of the microscopy algorithm. Data from a recent study in the DRC estimates the sensitivity of microscopy at 69.5% (95% CI = 63.0–75.3%) [15] and hence, 38% of cases in this study were detected following a positive LAMP test. In spite of adding LAMP to the algorithm, the risk of missing cases remains considerable, and hence suspects who are not confirmed by microscopy are re-tested with RDTs at three month intervals. Attempts to improve the accuracy of the LAMP method, such as by using real-time fluorescence readers or by designing novel primers, could be considered. For instance, including a second set of reaction accelerating primers was recently reported to improve test performance [40]. Due to an imperfect sensitivity of the HAT RDT, all severely sick RDT negative patients for whom no alternative diagnosis is reached are monitored by staff at local health facilities and by community health workers. This follow-up, along with following up and monitoring RDT positive suspects that are referred for microscopy, requires high-quality patient and data management at the facility level. In 2016, a new SMS based data capture system has been put in place, similar to programmes used in malaria logistics in Tanzania [41]. This system sends reminders by SMS to patients to go for testing, and so should improve patient compliance with the diagnostic requirements, as well as monitoring stock levels at health facilities.

The enhanced algorithm for passive screening and confirmatory diagnosis of gHAT described here can be deployed in gHAT foci in other countries that have under-utilised their public health infrastructure. The integrated nature of the strategy utilises existing health care infrastructure, and so has the appeal of minimising costs and management, by optimising deployment of resources and integrating HAT screening into routine healthcare activities. Studies would however be required to determine the costs and cost-effectiveness of implementing the strategy. In light of this, further work is being undertaken to estimate the total costs of this strategy and compare these costs to other algorithms for screening and diagnosis of gHAT.

## Supporting information

S1 Table Questionnaire used to characterise health facilities. (DOCX)

S2 Table Equipment installed and materials supplied to microscopy and LAMP facilities to perform parasitological and molecular testing for gHAT. (DOCX)

S1 Fig Number of serological tests (CATT and HAT RDT) performed in 4 health centres (Adjumani Hospital, Moyo Hospital, Omugo HCIV and Yumbe Hospital) from 2009 to 2015. (DOCX)
