# Supplementary material for: Enhanced passive screening and diagnosis for gambiense human African trypanosomiasis in north-western Uganda – Moving towards elimination
Source: PLoS One. 2017 Oct 12;12(10):e0186429. doi: 10.1371/journal.pone.0186429 (PMC5638538; doi:10.1371/journal.pone.0186429)
Supplement: S1 Table — (DOCX) [file pone.0186429.s001.docx]

**S1 Table:** questionnaire used to characterise health facilities

| Name and title of respondent |  |
| --- | --- |
| Date of survey (DD/MM/YYYY) |  |
| Settlement (Village) Name (official) |  |
| Name of 1st administrative level (Province) |  |
| Name of 2nd administrative level (State) |  |
| Name of 3rd administrative level (County) |  |

**Details of facility**

| Name (official) of health facility |  |
| --- | --- |

Geogrpahic coordinates (WGS84) of the health facility (in decimal degrees):

| Latitude |  |
| --- | --- |
| Longitude |  |

| Estimated catchment population |  |
| --- | --- |
| Estimated number of households in the catchment population |  |
| Year of estimated catchment population |  |
| Type of health facility | Select ONE:  National referral hospital  Regional referral hospital  District hospital  Health Centre IV  Health Centre III  Health Centre II  Other (Specify): |

| Records (History) of previous sleeping sickness cases | |
| --- | --- |
| 2012 |  |
| 2011 |  |
| 2010 |  |
| 2009 |  |

**Capacity of the health facility**

| Human resource capacity (numbers): | |
| --- | --- |
| Medical doctor |  |
| :Clinical officer |  |
| Health officer |  |
| Nurses / nurse assistant |  |
| MCH |  |
| Lab. Technician |  |
| Lab. Assistant |  |
| Microscopist |  |
| CHW / TBA / VHT |  |
| Others (specify): |  |

| Laboratory capacity (functional equipment available): | |
| --- | --- |
| Electric light microscope | Yes  No |
| Natural light microscope | Yes  No |
| Centrifuge | Yes  No |

| Laboratory consumables (available): | |
| --- | --- |
| Microscope slides | Yes  No |
| Capillary tubes | Yes  No |
| mAECT | Yes  No |
| Field’s / Giemsa stain | Yes  No |
| CATT test | Yes  No |

| Is sleeping sickness treatment available? | Yes  No |
| --- | --- |

*If ‘****No****’ then:*

| How far is it to the nearest facility with treatment capacity |  |
| --- | --- |
| Name the facility |  |

**Infrastructure**

| Energy source | Select ALL that apply:  Hydro-electricity  Thermal generator  Solar panels  No electricity |
| --- | --- |

| Water source | Select ALL that apply:  Piped water  Rain water  Bore hole  River / lake / well |
| --- | --- |

| Communications | Select ALL that apply:  Landline (record number)  Cell phone (record number)  Computer  Internet |
| --- | --- |
