# Supplementary material for: Enhanced passive screening and diagnosis for gambiense human African trypanosomiasis in north-western Uganda – Moving towards elimination
Source: PLoS One. 2017 Oct 12;12(10):e0186429. doi: 10.1371/journal.pone.0186429 (PMC5638538; doi:10.1371/journal.pone.0186429)
Supplement: S2 Table — (DOCX) [file pone.0186429.s002.docx]

**S2 Table:** Equipment installed and materials supplied to microscopy and LAMP facilities to perform parasitological and molecular testing for gHAT.

|  | **Equipment** | **Consumables** |
| --- | --- | --- |
| **All facilities performing confirmatory testing by microscopy** | Primo Star iLED microscope | Microscope slides and coverslips |
|  | Haematocrit centrifuge | Acridine orange stain |
|  | Bench centrifuge | RBC lysis solution |
|  | Capillary tube reading chamber | Falcon tubes 15 ml |
|  | Micropipettes 1000 ul | Pipette tips |
|  | Micropipettes 100 ul | Plastic pasteur pipettes |
|  | Micropipettes 10 ul | Plasticine |
|  | Solar installation | Heparinized tubes |
|  |  | Capillary tubes |
|  |  | Filter papers |
|  |  | Sealable pouches |
|  |  | Silica gel |
| **Additional equipment for LAMP facilities** | LAMP incubator | LAMP kits |
|  | Microcentrifuge centrifuge | Microcentrifuge tubes |
|  | Water bath | Centrifuge tubes |
|  | Micropunch (7 mm) | PCR grade water |
|  |  | SDS solution |
|  |  | PBS tablets |
