# Supplementary material for: Enhanced passive screening and diagnosis for gambiense human African trypanosomiasis in north-western Uganda – Moving towards elimination
Source: PLoS One. 2017 Oct 12;12(10):e0186429. doi: 10.1371/journal.pone.0186429 (PMC5638538; doi:10.1371/journal.pone.0186429)
Supplement: S1 Fig — (DOCX) [file pone.0186429.s003.docx]

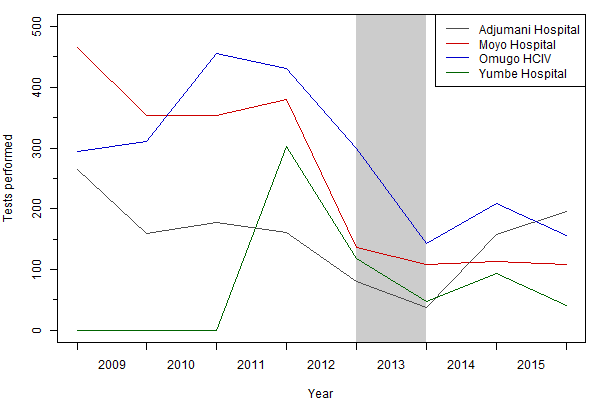


**S1 Fig**: Number of serological tests (CATT and HAT RDT) performed in 4 health centres (Adjumani Hospital, Moyo Hospital, Omugo HCIV and Yumbe Hospital) from 2009 to 2015.
